# Supplementary material for: Modeling glioblastoma heterogeneity as a dynamic network of cell states
Source: Mol Syst Biol. 2021 Sep 16;17(9):e10105. doi: 10.15252/msb.202010105 (PMC8444284; doi:10.15252/msb.202010105)
Supplement: Supplementary file 6 — Source Data for Figure 5 [file MSB-17-e10105-s004.zip › Figure5A_sourcedata/GSEA_3017/hallmarks_stateC.GseaPreranked.1621934520804/HALLMARK_TNFA_SIGNALING_VIA_NFKB.html]

Details for gene set HALLMARK\_TNFA\_SIGNALING\_VIA\_NFKB[GSEA]

|  || Dataset | state13017 |
| Phenotype | NoPhenotypeAvailable |
| Upregulated in class | na\_neg |
| GeneSet | HALLMARK\_TNFA\_SIGNALING\_VIA\_NFKB |
| Enrichment Score (ES) | -0.48015895 |
| Normalized Enrichment Score (NES) | -2.4452186 |
| Nominal p-value | 0.0 |
| FDR q-value | 0.0012142858 |
| FWER p-Value | 0.001 |
Table: GSEA Results Summary

  

Fig 1: Enrichment plot: HALLMARK\_TNFA\_SIGNALING\_VIA\_NFKB      
 Profile of the Running ES Score & Positions of GeneSet Members on the Rank Ordered List

  

| PROBE | GENE SYMBOL | GENE\_TITLE | RANK IN GENE LIST | RANK METRIC SCORE | RUNNING ES | CORE ENRICHMENT || 1 | SAT1 |  |  | 241 | -0.262 | -0.4094 | No |
| 2 | SOCS3 |  |  | 274 | -0.270 | -0.4464 | No |
| 3 | PLAU |  |  | 294 | -0.274 | -0.4598 | Yes |
| 4 | PHLDA1 |  |  | 295 | -0.274 | -0.4395 | Yes |
| 5 | NAMPT |  |  | 306 | -0.280 | -0.4365 | Yes |
| 6 | CSF1 |  |  | 320 | -0.285 | -0.4386 | Yes |
| 7 | SERPINE1 |  |  | 323 | -0.286 | -0.4210 | Yes |
| 8 | TUBB2A |  |  | 336 | -0.289 | -0.4209 | Yes |
| 9 | PPP1R15A |  |  | 342 | -0.293 | -0.4081 | Yes |
| 10 | TNFAIP6 |  |  | 359 | -0.301 | -0.4143 | Yes |
| 11 | PMEPA1 |  |  | 375 | -0.308 | -0.4182 | Yes |
| 12 | FOS |  |  | 377 | -0.309 | -0.3971 | Yes |
| 13 | RHOB |  |  | 391 | -0.316 | -0.3968 | Yes |
| 14 | GADD45A |  |  | 405 | -0.323 | -0.3960 | Yes |
| 15 | LDLR |  |  | 419 | -0.334 | -0.3943 | Yes |
| 16 | MYC |  |  | 421 | -0.336 | -0.3712 | Yes |
| 17 | SLC2A3 |  |  | 422 | -0.336 | -0.3464 | Yes |
| 18 | KLF6 |  |  | 429 | -0.339 | -0.3319 | Yes |
| 19 | B4GALT5 |  |  | 433 | -0.342 | -0.3119 | Yes |
| 20 | VEGFA |  |  | 436 | -0.345 | -0.2900 | Yes |
| 21 | PTPRE |  |  | 469 | -0.372 | -0.3193 | Yes |
| 22 | HES1 |  |  | 470 | -0.373 | -0.2917 | Yes |
| 23 | HBEGF |  |  | 480 | -0.386 | -0.2790 | Yes |
| 24 | TSC22D1 |  |  | 492 | -0.400 | -0.2690 | Yes |
| 25 | FJX1 |  |  | 501 | -0.418 | -0.2522 | Yes |
| 26 | TGIF1 |  |  | 502 | -0.421 | -0.2210 | Yes |
| 27 | JUN |  |  | 503 | -0.425 | -0.1895 | Yes |
| 28 | F3 |  |  | 505 | -0.432 | -0.1593 | Yes |
| 29 | IRS2 |  |  | 516 | -0.444 | -0.1442 | Yes |
| 30 | CEBPD |  |  | 523 | -0.455 | -0.1211 | Yes |
| 31 | PLPP3 |  |  | 528 | -0.466 | -0.0937 | Yes |
| 32 | RCAN1 |  |  | 544 | -0.505 | -0.0830 | Yes |
| 33 | TNC |  |  | 564 | -0.572 | -0.0745 | Yes |
| 34 | CD44 |  |  | 575 | -0.659 | -0.0434 | Yes |
| 35 | CCL2 |  |  | 595 | -1.066 | 0.0018 | Yes |
Table: GSEA details [plain text format]

  

Fig 2: HALLMARK\_TNFA\_SIGNALING\_VIA\_NFKB: Random ES distribution      
 Gene set null distribution of ES for **HALLMARK\_TNFA\_SIGNALING\_VIA\_NFKB**
